# The major acute phase proteins of bovine milk in a commercial dairy herd

**DOI:** 10.1186/s12917-015-0533-3

**Published:** 2015-08-15

**Authors:** Funmilola Clara Thomas, Mary Waterston, Peter Hastie, Timothy Parkin, Hayley Haining, Peter David Eckersall

**Affiliations:** Institute of Biodiversity, Animal Health and Comparative Medicine, College of Veterinary Medical and Life Sciences, University of Glasgow, Bearsden Rd, Glasgow, G61 1QH UK; Institute of Infection Immunity and Inflammation, College of Veterinary Medical and Life Sciences, University of Glasgow, University Avenue, Glasgow, G12 8QQ UK; School of Veterinary Medicine, University of Glasgow, Bearsden Road, Glasgow, G61 1QH UK

**Keywords:** Haptoglobin, Serum amyloid A, C-reactive protein, Somatic cell counts, Bovine mastitis

## Abstract

**Background:**

Milk acute phase proteins (APP) have been identified and show promise as biomarkers of mastitis. However analysis of their profile in dairy cows from a production herd is necessary in order to confirm their benefits in mastitis diagnosis. The profiles of milk haptoglobin (Hp), mammary associated serum amyloid A3 (M-SAA3) and C-reactive protein (CRP) were determined in 54 composite milk (milk from all functional quarters of a cow’s udder collected in a common receptacle) samples (CMS) from a commercial dairy farm. Milk Hp was also determined in individual quarter milk (milk from a single udder quarter) samples (QMS) (*n* = 149) of the cows.

An ELISA was developed and validated for the determination of milk Hp while commercial kits were used for M-SAA3 and CRP assay respectively. Composite milk APP results were compared with cow factors including parity, stage of lactation, percentage protein and fat as well as somatic cell counts (SCC).

**Results:**

Composite milk Hp ranged from <0.4–55 μg/ml with a median of 3.5 μg/ml; composite milk M-SAA3 ranged from <0.6–50 μg/ml and had a median of 1.2 μg/ml, while CRP ranged from <1.80–173 ng/ml and had a median of 24.6 ng/ml. Significant correlations were found between composite SCC and Hp (*P*-value <0.009) as well as parity and Hp (*P* < 0.009), but not between M-SAA3 and SCC, M-SAA3 and Hp, M-SAA3 and CRP or M-SAA3 and parity. Milk CRP was correlated with % fat (*P* = 0.002) and % protein (*P* = 0.001) of the milk samples. The lack of correlation of SCC with the M-SAA3 and CRP could result from these APP being more sensitive to intra-mammary infection than SCC. Quarter milk Hp had a range of <0.4–420 μg/ml with a median value of 3.6 μg/ml, with 92 % of samples below 20 μg/ml.

**Conclusion:**

Baseline values of Hp, M-SAA3 and CRP were established in composite milk from cows with normal SCC on the dairy farm. Parity was recognized as a possible confounding factor when diagnosing mastitis using Hp. The value of the APP, Hp, M-SAA3 and CRP as substitutes or to complement SCC in indicating udder inflammation, was demonstrated.

## Background

Mastitis, inflammation of the mammary gland, is an expensive and prevalent condition in most dairy farms, contributing to massive economic losses to the industry. The impact of mastitis on dairy farms worldwide continues to rise, as it is the leading contributor to economic losses in the industry and represents a major welfare concern for the dairy cow [1]. It affects the composition, quality, yield and processing property of milk [2]. The problem of mastitis is compounded by the incidence of subclinical mastitis (SM) which is a form of the disease where signs of inflammation (systemically and locally) are absent [3]. Consequently, an inability to readily recognise and diagnose animals with SM occurs, leading to a delay in treatment and control of infections thus allowing a possible spread to other uninfected quarters and cows.

Somatic cells such as neutrophils increase greatly in the mammary tissue and milk during intra mammary infections (IMI), [4] so somatic cell count (SCC) values are used as indicators of mastitis usually being determined in diagnostic laboratories. SCC is currently the most common way to diagnose SM on dairy farms [3, 5]. However, SCC is affected by factors other than IMI, such as physiological status, seasonal variation and has also been shown to persist in milk long after the removal of infectious causes of mastitis from milk [5]. It is also difficult to adapt to on line testing of milk, hence alternative indices for SM detection are required. Among alternatives to the SCC, the California mastitis test (CMT) is a cow side test that was developed by Schalm and Noorlander [6] as an indirect measure of somatic cells in milk but suffers from a lack of sensitivity and reproducibility.

Electrical conductivity (EC) of milk, infrared thermography (IRT) and assay of milk enzymes such as N-acetyl-β-D-glucosaminidase (NAGase) as well as lactate dehydrogenase have also been employed for the detection of mastitis in milk; however, these tests are not always sensitive and specific for the disease [5].

The use of APP for the diagnosis and prognosis of inflammatory conditions has been exploited for a long time in human medicine and their assay has become a significant diagnostic tool in veterinary medicine [7]. APP are proteins produced mainly in the liver and released into serum, following stimulus by inflammatory cytokines; particularly interleukin-1 (IL-1), interleukin-6 (IL-6), and tumour necrosis factor alpha (TNFα), in the acute phase response (APR) as part of the innate immune response [8, 9].

Major APP are those whose concentration increases up to and over 100 fold following inflammation. In cattle the major APP are SAA and Hp, while α-l acid glycoprotein (AGP) increases 2–3 folds in chronic inflammation [9] and is considered a moderate APP. Milk, uterine fluid, synovial fluid and nasal secretion are other biological samples other than serum, where APP have been detected [10–13]. The presence of APP in tissue or organ (fluids) could be considered a more specific indicator of inflammation localized in that organ especially when there is no corresponding rise in serum APP [14]. Therefore milk APP could be specific and sensitive indicators of IMI [15].

Haptoglobin is a tetrameric protein made up of two α (~20 kDa) and two β chains (~35 kDa) linked by disulphide bonds. It exists in bovine serum in polymeric forms and binds to free haemoglobin (Hb), transporting it from the blood to the liver where Hb is recycled [16]. The source of Hp in milk during mastitis has been demonstrated to be either one or a combination of; migrating neutrophils, mammary gland tissue, somatic cells or serum leakage [17–21].

Haptoglobin can be assayed directly by antibody detection using immunoassays such as enzyme linked immunosorbent assay (ELISA) and single radial immunodiffusion [22], as well as indirectly by measuring the activity of Hb peroxidase exploiting the high affinity binding of Hp to free Hb [23]. Direct immunoassays using antibodies have an advantage of higher sensitivity than assays using Hb binding.

Serum amyloid A is an APP that has several protein species and is known to be primarily produced in the liver in response to acute phase stimulus. Studies have also shown it is produced from many other extra-hepatic tissues including the mammary gland [24–26] It is a small protein of molecular weight between 10–17 kDa with about 112 amino acid residues [27], its main isoforms are SAA1, SAA2 and SAA3 with the SAA1 and SAA2 produced in the liver while SAA3 is produced in extra-hepatic sites and the type predominantly found in milk and has been called mammary associated amyloid A (M-SAA3) [24].

C-reactive protein is a pentameric protein (5 identical subunits linked together non-covalently) with a molecular weight of about 115 kDa secreted from the liver in response to cytokine stimulation and known to play roles in activating phagocytosis by binding to the phosphocholine portion of pathogens or dying cell membranes, complement activation, opsonisation of pathogens and binding to immunoglobulin receptors [28]. C-reactive protein has been generally considered a minor APP in bovine due to minimal changes observed in its concentrations in serum of cattle during inflammatory conditions [29], however studies have demonstrated its potential as a parameter of mastitis in milk [30–32] but insufficient data exists on the profile of CRP in healthy versus mastitis milk and on the correlation of CRP with other mastitis parameters, notably SCC and milk APP.

The APP, Hp and M-SAA3 have been demonstrated to correlate with SCC and bacteriology in cases of subclinical and clinical mastitis of natural [9, 14, 33] and experimental [25, 26, 34–36] origins, thus, can be termed diagnostic markers of mammary inflammation.

There have been studies on the measurement of APP such as Hp in milk, using rapid and on-farm assay-format that could enhance their usefulness in diagnosis of mastitis [37, 38]. However, numerous gaps exist in our knowledge of the potential use of Hp, M-SAA3 and CRP assays for detection of mastitis. These include, ascertaining the range of the concentrations of milk APP in commercial dairy farms in order to establish basal concentrations and suitable cut off points and reference values for discriminating infections (clinical or subclinical) from health as well as distinguishing severity of infections. It is important to determine these in composite milk samples (CMS) as well as in quarter milk samples (QMS) with the former being the sample most commonly collected for tests such as SCC analysis. Furthermore, it is essential to determine how physiological conditions affecting the cow, notably parturition, influences the range of values for major APP in milk and at what point APP become useful in indicating new IMI in the periparturient period.

Our hypothesis was that the basal concentration range of these APP (in non-infected milk samples) would be significantly different from that of infected milk and that significant correlations would exist between SCC levels and the APP in milk.

It was therefore the objective of this study to establish a reference range for APP in a commercial herd where SM is likely to occur. In order to achieve this aim, an ELISA for the measurement of Hp in milk was developed and optimized.

Haptoglobin was assayed using the ELISA in QMS and CMS to assess the level of variability between quarter and composite milk. Mammary associated serum amyloid A3 and CRP were assayed in composite milk of the production herd using commercial ELISAs. Correlations between composite milk APP and commonly assessed milk traits (SCC, protein and fat percentage) and cow factors (parity and stage of lactation) were explored.

## Methods

### Sample collection

Milk samples were collected from Holstein–Friesian cows at the University of Glasgow Cochno Farm and Research Centre, between the periods of September, 2012 to June, 2013. Cows were milked twice daily and fed in-parlour concentrates and total mixed ration (TMR- silage plus concentrates). Lactation number ranged from 1 to 10. Calving occurs year round and cows sampled were at different stages of lactation including early, mid and late lactation. Health statuses of cows were confirmed by history of routine veterinary check-ups on the cows and all were assessed as being healthy and the use of the animals for study was approved by the Ethics Committee of the School of Veterinary Medicine, University of Glasgow.

Composite milk samples were collected during usual morning milking from each milk-producing cow on the farm, as used for routine monthly SCC recording. Teats were disinfected using an iodine based pre-milking teat dip, followed by the removal of the first few jets of milk and then the application of teat cups of the milking machine onto the functional teats of each cow. Milk samples were collected from a milk tube linked to the milk line from each cluster of the milking machine and transferred into sterile tubes. Aliquots of this in-line composite sample were made; a 5 ml aliquot was obtained for APP measurement for each cow (*n* = 54).

Individual quarter milk samples (QMS) were also obtained from each cow. After disinfection, the first jets of milk were discarded then approximately 40 ml of milk was collected from each quarter into separate sterile 50 ml Falcon tubes, these were used for Hp determination in order to ascertain the relationship between QMS and the CMS from the same cow. All APP assays were carried out using whole milk samples.

### Milk Hp ELISA

Purified rabbit anti bovine Hp IgG (Life Diagnostics *Inc.*, West Chester, USA) was conjugated to alkaline phosphatase (Innova biosciences, Cambridgeshire UK) according to the manufacturer’s instructions and used in a sandwich ELISA procedure.

Unconjugated rabbit anti bovine Hp IgG (Life Diagnostics *Inc.*, West Chester, USA) was used as a capture antibody by incubating 100 μl of a dilution of the rabbit anti bovine Hp at 0.125 μg/ml in coating buffer (0.05 M NaHCO_3_, pH 9.6) in each well of a Nunc-Maxisorp 96 MicroWell™ plate (Nunc International, Rochester, New York USA) overnight at 4 °C. Wells were washed using 0.02 M Tris–HCl pH 7.4 with 0.05 % (v/v) Tween-20 (assay/wash buffer) 4 times (x). Blocking of unoccupied sites on the well was achieved by addition of 250 μl of 10 % (w/v) dried milk protein (in wash buffer) per well and incubating for 60 min at 37 °C. After washing, standard bovine Hp (1.64 mg/ml, Life Diagnostics Inc., West Chester, USA) was used for standards. A serial dilution in assay/wash buffer was made to give standards of Hp from 1025 ng/ml to 8 ng/ml. Milk samples were also diluted at 1:800 in assay/wash buffer. 100 μl of each standard bovine haptoglobin and milk sample were added in triplicate to wells and incubated at 37 °C for 60 min with gentle shaking.

Wells were washed 4 x, and 100 μl of the alkaline phosphatase-conjugated antibody diluted at a dilution of 1:10,000 in wash buffer was dispensed into each well of the ELISA plate and incubated at room temperature for 60 min with gentle shaking. After washing, substrate solution, BluePhos® Microwell phosphatase substrate system (KPL laboratories, Inc., Maryland USA) was made up according to manufacturer’s instructions and 100 μl was added into each well for colour development for approximately 10 min. APstop™ (KPL laboratories *Inc*. USA) solution was dispensed by adding 100 μl per well, to stop further colour development after the optimum was reached.

The absorbance was read at 595 nm using a FLUOstar OPTIMA plate reader (BMG Labtech Ltd., Bucks, United Kingdom) and the results analysed and calculated using the associated FLUOstar OPTIMA Software V1.32 R2. A 4 parameter logistics (4PL) logarithm-linear scale curve was used to plot the standards. Sample Hp concentrations were interpolated from the linear portion of the standard curve.

The assay was validated with the inter assay precision determined by calculating coefficient of variance (CV) of ten repeats of high and low quality control (QC) samples in different plates and on separate dates, while intra assay precision was determined by calculating the mean CV of 40 samples run in duplicates on a single ELISA plate. Accuracy was evaluated by percentage recovery of standard bovine Hp (Life Diagnostics *Inc*., West Chester USA) in spiked milk samples. Specificity was assessed by western immunoblotting of milk samples (± Hp high, low spiked samples) as described in Braceland et al. [39] using a rabbit anti bovine haptoglobin (Life Diagnostics *Inc.,* West Chester, USA) at a dilution concentration of 1 in 10,000. Sensitivity was assessed by determining the limit of detection (LOD) by calculating the Hp concentration at +3 standard deviations from the mean of 4 blank samples (assay buffer).

### Mammary associated serum amyloid A3 Assay

M-SAA3 was determined using a commercial multispecies SAA ELISA kit (Tridelta Development Ltd, Wicklow, Ireland) according to manufacturer’s instruction as used in Eckersall et al. [26] with minor modifications; samples were diluted to a minimum dilution of 1:50, or 1:500. The absorbance of the ELISA was read at 450 nm using FLUOstar optima plate reader. The LOD of the assay was determined to be 0.6 μg/ml (calculated from mean of four blanks +3 standard deviations), while the intra-assay and inter-assay CVs were 7 % (mean CV of 40 samples assayed in duplicate) and 33 % (mean CV of 2 QC samples in 5 different assays) respectively.

### C-reactive protein assay

Cow C-reactive protein (CRP) ELISA kits for assay of milk CRP were supplied by Life Diagnostics *Inc*. (West Chester, USA). The assay was based on solid phase sandwich ELISA format, and comprised of a primary anti-bovine CRP antibodies immobilized to the wells of a 96-well microtitre plate and secondary antibodies against the anti-bovine CRP conjugated to horse radish peroxidase (HRP).

The assay for bovine milk CRP was carried out according to the manufacturer’s instructions; diluent buffer and wash buffer were prepared from the stock of 10 x and 20 x solution respectively, using milli Q water according to the manufacturer’s instruction.

CRP standard was reconstituted by adding 1 ml of the 1 x diluent buffer into the vial of lyophilized standard and vortexed vigorously. 14.25 μl of the reconstituted standard was added to 485 μl of diluent buffer to give the top standard with a concentration of 62.5 ng/ml. The top standard was then serially diluted to give 6 other standards with concentrations ranging from 62.5 ng/ml to 0.98 ng/ml. Plain diluent buffer was used as the blank (0 ng/ml). Milk samples were diluted initially at 1:250 in 1 x diluent buffer, but for samples with higher CRP concentrations, a dilution of 1:2000 was used, for samples with very low CRP concentration a lower dilution of 1:5 was used.

Diluted samples and standards were mixed thoroughly and 100 μl of each sample or standard was dispensed into duplicate wells of the 96-well microtitre plates provided. This was then incubated on an orbital microplate shaker at 150 rpm at room temperature for 45 min. Contents of the wells were then discarded and wells washed 5 x each using 300 μl of 1 x wash buffer per well. After ensuring all residual droplets in the wells were removed by striking plates onto absorbent paper, 100 μl of the secondary antibody-HRP conjugate was then dispensed into each well and incubated on the shaker at room temperature for 45 min. The wash step was repeated and 100 μl of TMB reagent (HRP substrate) was dispensed into wells and colour development was allowed to proceed for 20 min on the shaker at room temperature. The reaction was stopped by adding 100 μl of stop solution per well into the wells. Absorbance was read using a FLUOstar Optima plate reader at 450 nm within 15 min of stopping the reaction. A 4PL curve was used to plot the standards, and concentrations of samples were interpolated from the linear portion of the curve.

The LOD of the CRP assay was calculated from the mean plus 3 standard deviations of 4 blank samples while the intra-assay precision; mean CV of 30 samples assayed in duplicates in one ELISA plate and inter-assay; mean CV of 5 repeats of 2 QC samples.

### Somatic cell counts and milk data

Data of SCC, percentage fat and percentage protein in milk samples as well as lactation number (number of times cow had calved) and days in milk (DIM) of the cows were obtained from farm records (SCC, fat and protein percentage tests were carried out by the National Milk Records Company (NMR Co., Hillington-Park, Glasgow).

Although veterinary inspection did not identify observable clinical mastitis in the cows on the study there were raised levels of SCC in a number of samples. For analysis SCC were categorized into high (>200,000 cells/ml) and low (≤200,000 cells/ml), based on suggestions by Pantoja et al. [40]. Cut off values for SCC to determine subclinical mastitis have been a subject of debate [41], therefore in this study a second categorisation level for SCC was used based on suggestions of Schwarz et al. and Berglund et al. [42, 43] (healthy samples- SCC <100,000 cells/ml; SM samples -SCC 101,000–200,000 cells/ml; clinical mastitis (CM) samples-SCC >200,000 cells/ml). The APP distributions were compared between these various SCC categories.

### Statistical analyses

The statistical package for social sciences (SPSS) software (version 21) was used for statistical analyses. Comparison of each APP concentration in CMS (*n* = 54) between different groups of SCC; high (≥200, 000 cells/ml) or low (<200, 000 cells/ ml) were carried out using Mann-Whitney’s *U* test. Comparison of APP distribution in healthy, SM and CM SCC range as defined above were carried out using the independent Kruskal-Wallis test. Non-parametric correlation test (Spearman’s rho) was run to assess for correlations between each APP and milk parameters (SCC, % fat and % protein) and cow factors (parity and stage of lactation). Stage of lactation was determined from DIM as: 0–60 days = early lactation, 61–240 days = mid lactation; 241–305 days = late lactation). *P*-value was considered significant at <0.05. Stata® statistical package (version SE/12.1) was used to evaluate the receiver operating characteristic (ROC) and determine cut off of the APP in milk with varying levels of SCC.

## Results and discussion

In this study, the profile of major bovine APP, SAA and Hp as well as a minor bovine APP, CRP, in milk was determined in a commercial dairy farm, irrespective of the mastitis status of the cows. A reliable and specific sandwich ELISA, which was validated to be sufficiently sensitive and reproducible was developed and was useful in measuring concentration of Hp in quarter and composite milk samples. This assay for milk Hp using readily available antiserum to bovine Hp is a valuable method for measuring this APP in milk and will have application in future studies. The LOD for the assay was 0.4 μg/ml. The intra-assay and inter-assay precision were determined from coefficient of variance (CV) respectively and were 6 % and 27 % correspondingly. These CVs were considered to be acceptable for assay of Hp in milk where the changes in concentration of Hp can go from a rise of over 2000 %. A mean accuracy of 89.6 % and 96 % were determined through the recovery of Hp from spiked milk samples and through linearity of dilution of 3 different milk samples over a range of 3 dilution factors respectively. ELISAs as well as haemoglobin binding assays have been used for measuring Hp in serum samples however it is apparent that milk lactoperoxidase interferes with the Hb-Hp complex peroxidase activity, therefore immunoassays have been preferred for measuring Hp in milk [9, 17, 38]. The assay developed in this study had a lower or similar limit of detection than those previously reported [9, 17, 36] and was also able to measure baseline values of Hp in milk from low SCC cows similar to the assay described by Hiss et al. [17] which had a limit of detection in the same region (0.07 μg/ml).

Limit of detection of the M-SAA3 ELISA was <0.6 μg/ml, intra-assay CV was 7 % (mean CV of 40 samples assayed in duplicates) while inter-assay CV was 33 %. Dilutions of 1:50 or 1:500 were used depending on the concentration of M-SAA3 in the each milk sample.

Milk CRP had a LOD of 1.8 ng/ml, while the intra-assay precision was 4 % and inter-assay was 7 % and this novel ELISA was validated for use in assessment of the CRP concentration in bovine milk which should allow further study of the pathophysiology of this protein in mastitis..

Analysis of the composite milk samples from the farm demonstrated that, despite all cows being classed as healthy by inspection, there were a number of samples with elevated SCC (46.3 % and 31.5 % CMS had SCC above 100,000 and 200,000 cells/ml respectively). This is not an unusual finding and demonstrates the prevalence of SM on the farm but provides a means to determine if APP analysis could play a role in improving the detection or monitoring of IMI in the future.

### Composite milk APP

Descriptive statistics of CMS APP are presented in Table 1. Hp concentration of CMS ranged from <0.4–55.5 μg/ml with a median of 3.5 μg/ml.

**Table 1:** Descriptive statistics of all composite milk samples data. Acute phase proteins (Hp, M-SAA3 and CRP), somatic cell counts and other milk and cow data (SEM-Standard error of mean, SD- standard deviation)

	Hp (μg/ml)	MSAA3 (μg/ml)	CRP (ng/ml)	SCC (x 1000 cells/ml)	Parity	% fat	% protein	DIM (days)
Mean	6.97	3.87	32.64	485	3	11.75	3.47	222.20
SEM	1.47	1.08	5.00	159	0.28	7.55	.06	20.11
Median	3.46	1.17	24.56	96	3	4.28	3.44	188.50
SD	10.82	7.95	36.76	1170	2	55.50	.41	147.79
Minimum	<0.4	<0.6	<1.80	9	1	2.79	2.71	11.00
Maximum	55.46	50.13	172.47	6154	10	412.00	4.84	565.00

Acute phase proteins concentration was categorized in relation to SCC in two formats; firstly low SCC (<200,000 cells/ml) and high SCC (>200,000 cells/ml) to differentiate possible counts for CM from other cases (although all cows in the farm were clinically healthy). A value of 200,000 cells/ml has been suggested as a cut off point for separating healthy from mastitis affected milk [44]. Secondly, APP were also categorized to differentiate healthy from SM and CM with range of SCC <100,000 cells/ml (healthy), 101,000–200,000 cells/ml (SM) and >200,000 cells/ml (CM) as previously suggested [42, 43, 45]. Cut off values for SCC for use in diagnosis of mastitis has been a subject of debate [41] as it varies with different localities and type of milk samples (quarter, composite or bulk tank) [42, 46–48].

**Fig. 1:**
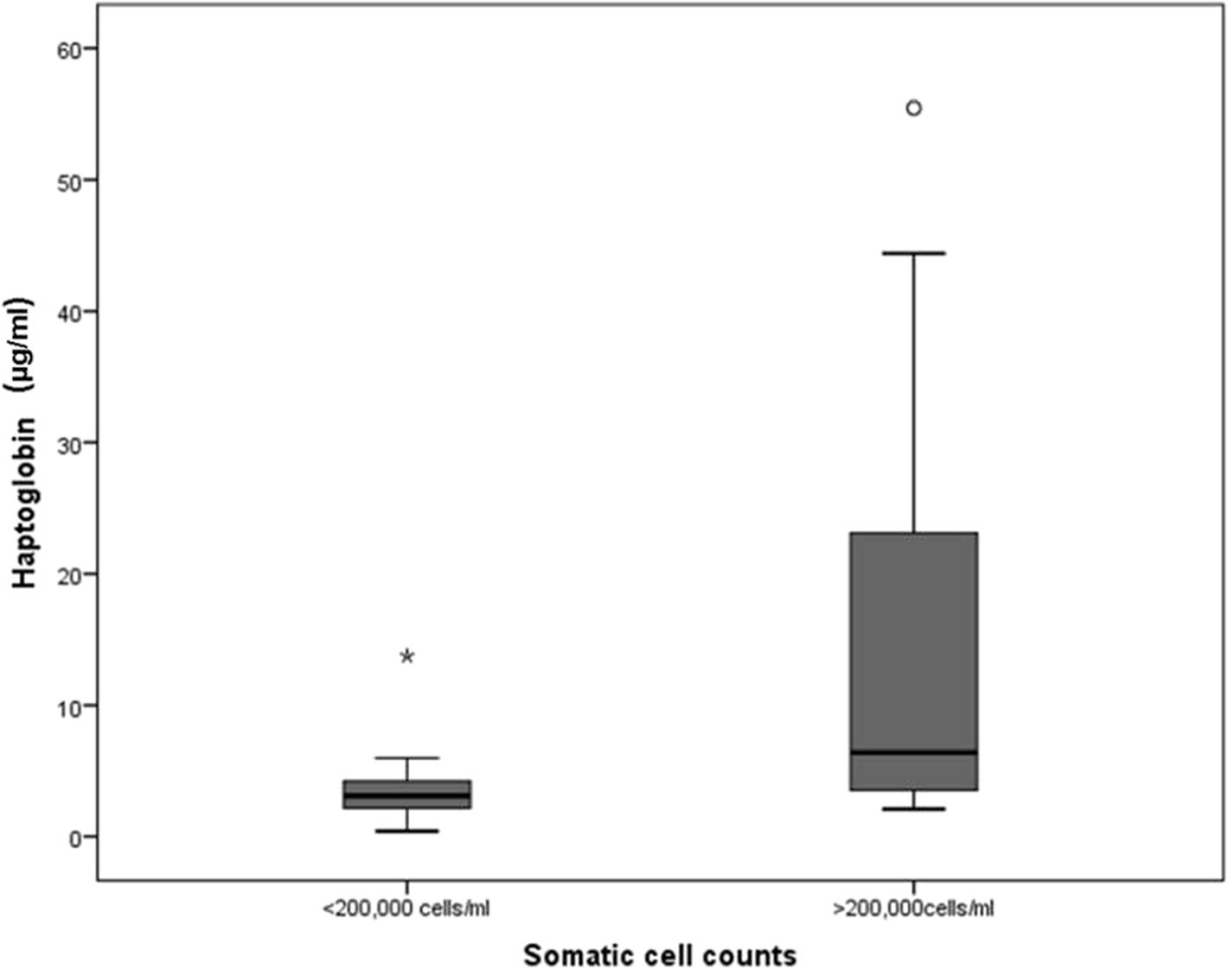
Boxplot showing Hp concentration (μg/ml) in two SCC categories of composite milk samples * indicates an extreme value (values greater than 3 interquartile range (IQR) away from 25^th^ or 75th percentile); IQR = 3^rd^ quartile -1st quartile (represented by the height of the box). ° indicates an outlier value (values greater than 1.5 interquartile range (IQR) away from 25^th^ or 75th percentile); IQR = 3^rd^ quartile -1st quartile (represented by the height of the box)

**Fig. 2:**
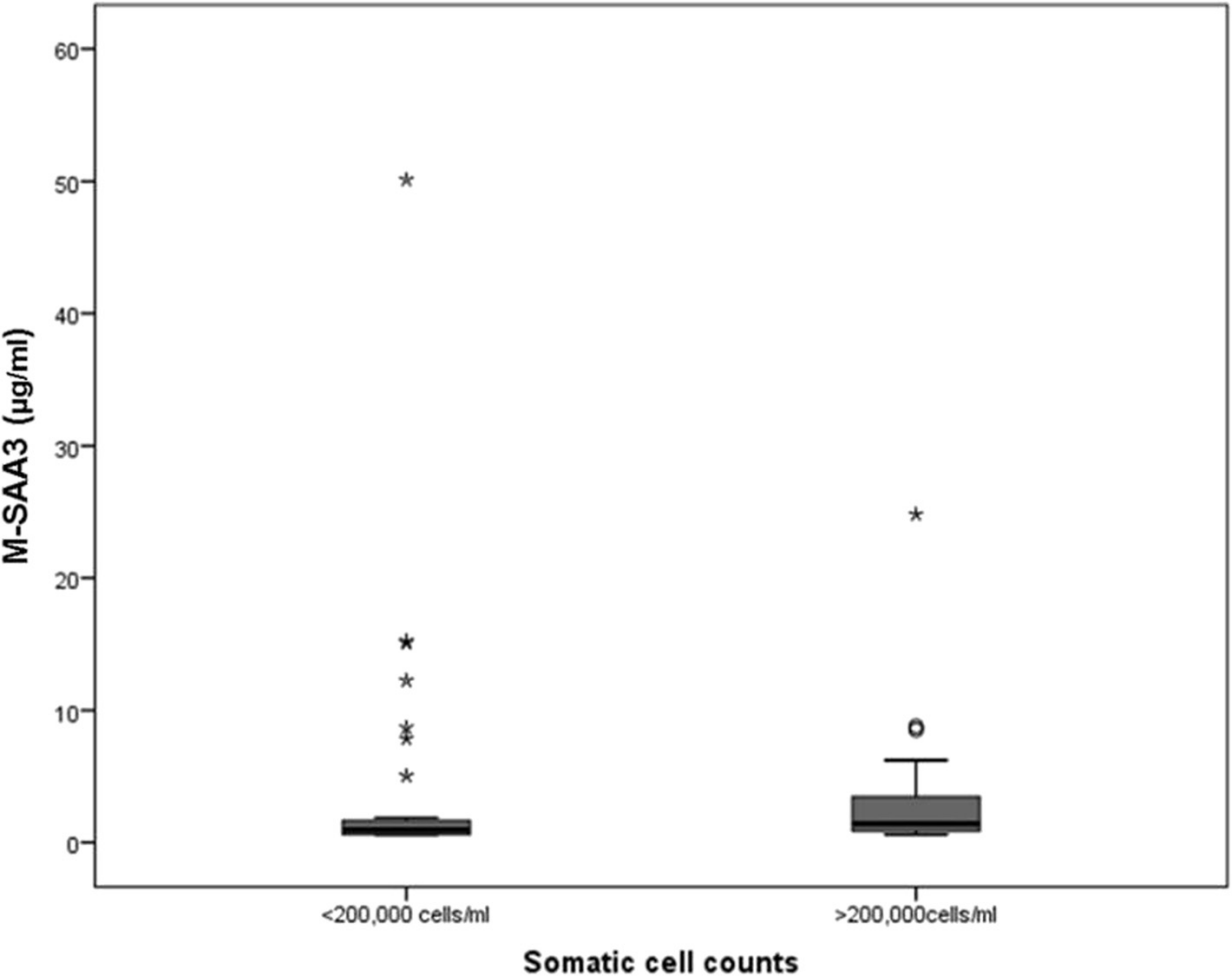
Boxplot showing M-SAA3 concentration (μg/ml) in the two SCC categories of composite milk samples * indicates extreme values (values greater than 3 interquartile range (IQR) away from 25^th^ or 75th percentile); IQR = 3^rd^ quartile -1st quartile (represented by the height of the box). ° indicates an outlier values (values greater than 1.5 interquartile range (IQR) away from 25^th^ or 75th percentile); IQR = 3^rd^ quartile -1st quartile (represented by the height of the box)

**Fig. 3:**
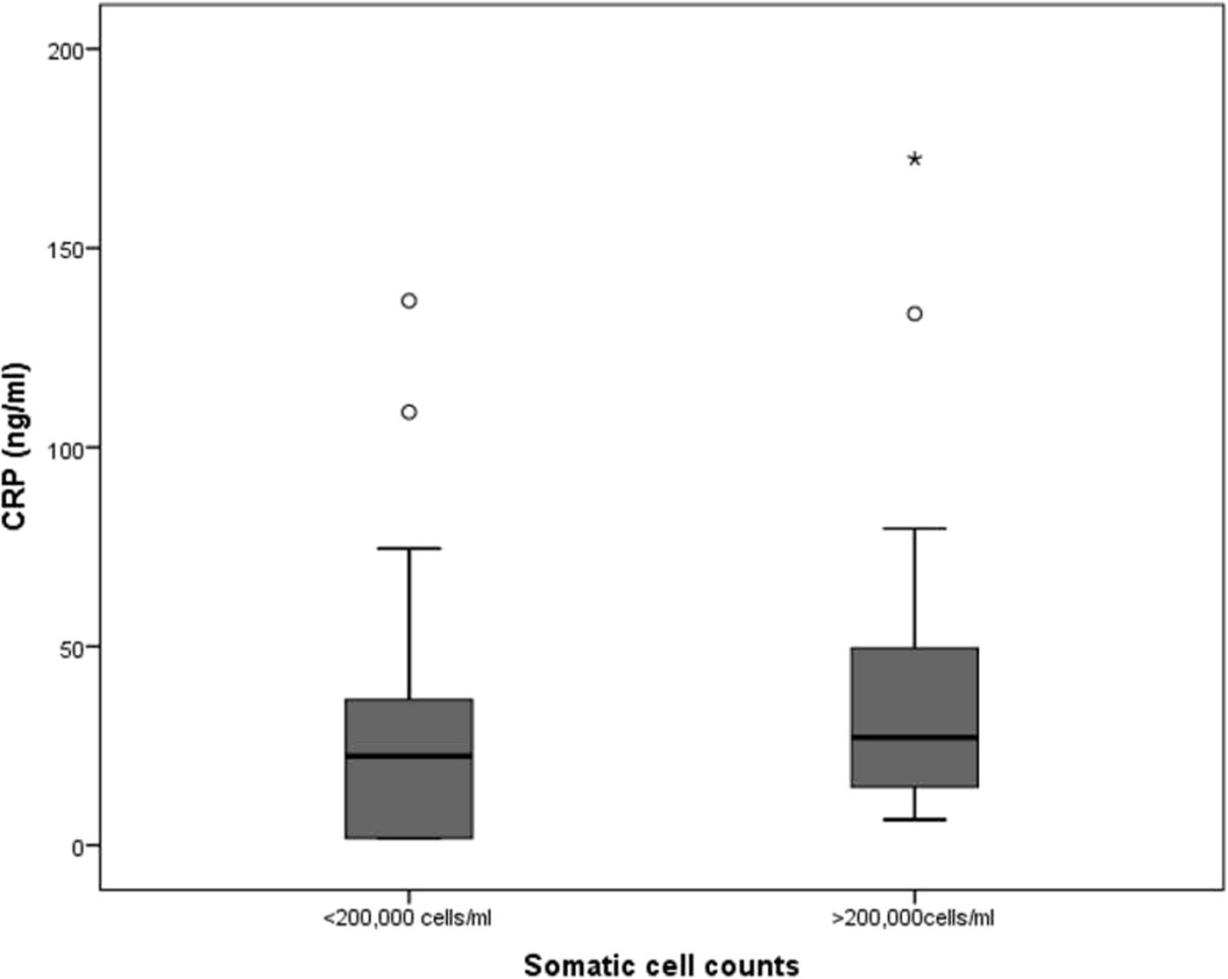
Boxplot showing CRP concentration (ng/ml) in the two SCC categories of composite milk samples * indicates an extreme values, (values greater than 3 interquartile range (IQR) away from 25^th^ or 75th percentile); IQR = 3^rd^ quartile -1st quartile (represented by the height of the box). ° indicates outlier values (values greater than 1.5 interquartile range (IQR) away from 25^th^ or 75th percentile); IQR = 3^rd^ quartile -1st quartile (represented by the height of the box)

Significant differences were observed in the Hp concentrations of the SCC high (>200,000 cells/ml) and low (≤200,000 cells/ml) categories (*P* = 0.001), Fig. 1 shows box and whiskers plots of Hp in the two SCC categories. There were no significant differences in the M-SAA3 and CRP values of milk from high (>200,000 cells/ml) or low (≤200,000 cells/ml) SCC groups (box plots shown in Figs. 2 and 3 respectively). In this study, we used these SCC values as cut off value for discriminating a high from low SCC in composite milk. No significant variations in the APP of the second set of SCC categories healthy (≤100,000 cells/ml), SM (101,000–200,000 cells/ml) and CM (>200,000 cells/ml) was observed, descriptive statistics of each APP in these categories are given in Table 2. This may be due to the small number of samples per group following this categorisation. The lack of statistical significance between the groups based on SCC cut off values in M-SAA3 and CRP and with the second set of SCC categories may be due to elevated concentration of the APP in the ‘healthy’ group which are selected on the basis of SCC result. It is possible that the APP are more sensitive to IMI than SCC which results in elevation of the APP when SCC is not affected. Raised levels of the APP indicate that there is an on-going cytokine mediated inflammatory response and may in practise give a more sensitive indication to the presence of mastitis than the currently used SCC test and it may be that combining results from all 3 APP would provide more diagnostic information.

**Table 2:** The descriptive statistics of Hp, M-SAA3 and CRP for healthy, SM and CM range of SCC in composite milk samples

Somatic cell counts (cells/ml)	Hp (μg/ml)		M-SAA3 (μg/ml)		CRP (ng/ml)	
	Median	Range	Median	Range	Median	Range
Healthy (<100 000 cells/ml); *n* = 29	2.96	<0.4–13.74	0.6	<0.6–50.13	22.40	<1.8–136.72
SM (101 000–200 000 cells/ml); *n* = 8	4.02	<0.4–5.28	0.6	<0.6	30.63	<1.8–108.84
CM (>200 000 cells/ml); *n* = 17	6.40	2.08–55.46	0.6	<0.6–24.81	27.12	6.44–172.46

Stata® statistical package was used to calculate reference values for Hp, M-SAA3 and CRP in relation to high and low SCC categories (>200,000 and <200,000 cells/ml). The area under curve (AUC) of the receiver operator curve (ROC) for M-SAA3 and CRP were not adequately sensitive and specific, but for Hp optimal cut off was determined to be 7.9 μg/ml (AUC = 0.78, odds ratio = 1.3) with an average specificity of 94.6 % but a poor sensitivity average of 52.9 %.

To obtain reference values that differentiate healthy from SM samples of CMS based on SCC values >100,000 cells/ml, a ROC analysis was performed giving an AUC of 0.75 was obtained for Hp with cut off value of 3.5 μg/ml (sensitivity of 74 % and specificity of 69.44 %). The values found here were in the same order as those of Hiss et al. [17], Safi et al. [49] Åkerstedt et al. [3].

Significant correlations were found between Hp and SCC (*P* < 0.01), Hp and parity (*P* < 0.05) and SCC and parity (*P* < 0.05). M-SAA3 was not significantly correlated to Hp (*P* = 0.406), SCC (*P* = 0.129) or parity (*P* = 0.293) in composite milk samples. C-reactive protein was also not correlated to Hp, M-SAA3 or SCC. However, we found CRP to be significantly correlated to the % fat and % protein (*P* = 0.002 and 0.001 respectively).

The correlation of Hp to SCC is expected as milk Hp has been shown to originate at least in part from somatic cells such as neutrophils [17, 19, 20] whereas M-SAA3 was shown to be synthesized in the mammary epithelial cells [49] and this fact may explain where M-SAA3 was not seen to correlate with SCC considering their different origins.

### Quarter milk sample Hp

**Fig. 4:**
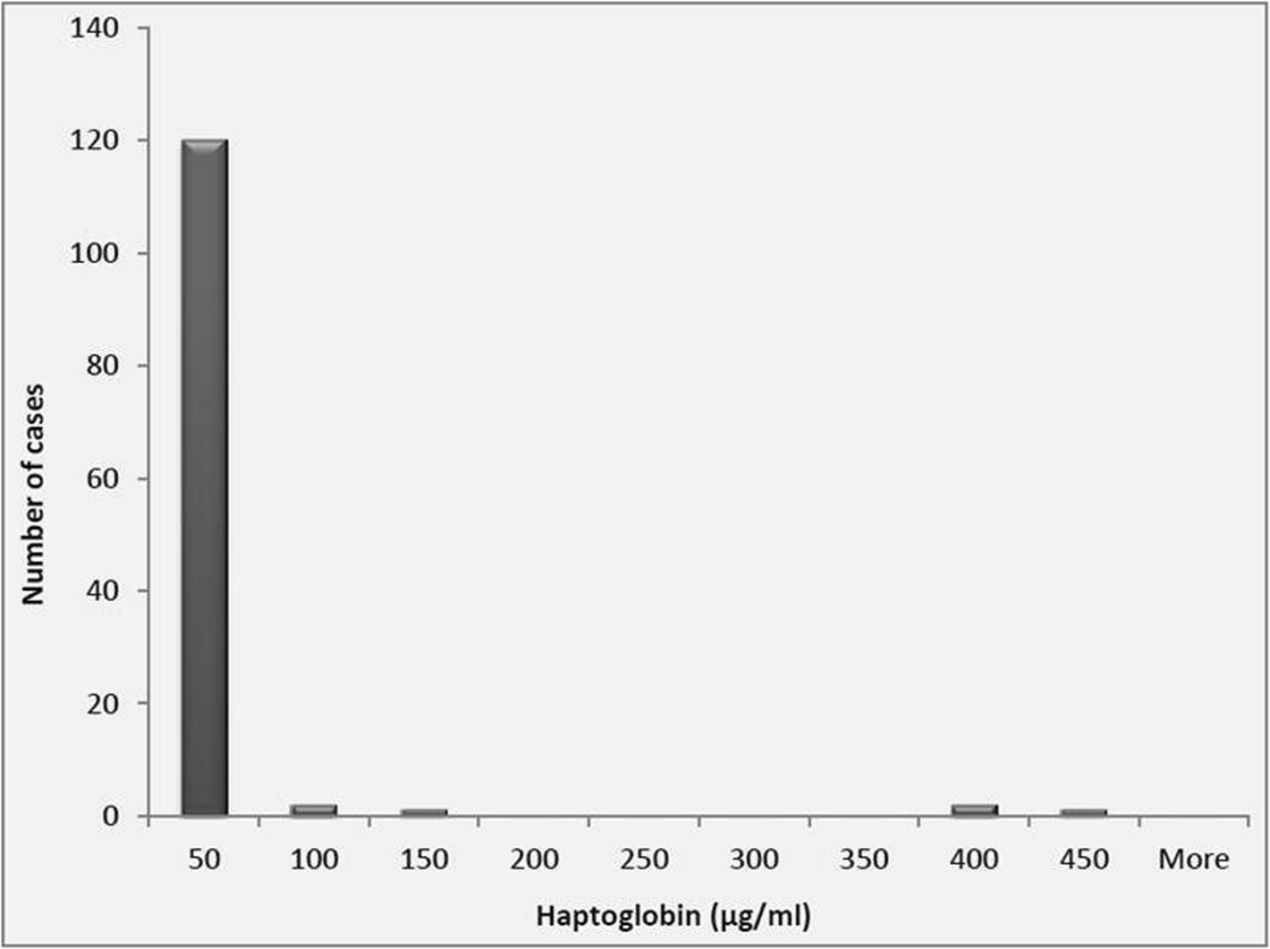
Distribution of milk Hp concentration in quarter milk samples (*n* = 149). Median-3.60 μg/ml, range <0.4–420 μg/ml

Haptoglobin from individual QMS from cows in the herd were not normally distributed with a higher percentage of samples falling within the low Hp category, Hp concentration in QMS ranged from <0.4–420 μg/ml with a median of 3.6 μg/ml, shown in Fig. 4. The higher maximum concentration of quarter milk samples than the composite samples highlights the effect of dilution by milk of non-infected quarters on infected quarters’ parameters which has been reported by several authors for example for SCC by Åkerstedt et al. [3]. It would therefore be in the interest of greater accuracy of detecting IMI and infected quarters to use quarter-milk samples for assay of APP.

Quarter milk SCC have been recognized to provide additional sensitivity in detecting mastitis glands [42], in this study however, only the CMS SCC were recorded, therefore QMS Hp could not be compared with SCC.

### Milk acute phase protein and udder health

The potential for use of APP in milk as markers of mastitis has been suggested since they were first described in milk [9] but in order to become recognised as an effective biomarker of this economically important condition it is necessary to establish the criteria for use. This investigation has been on a small scale and on only one farm and is therefore limited but it provides sufficient information to suggest that larger scale trials are warranted. Here it has been shown that Hp is the APP that provides the closest results to SCC with a significant correlation between their results. It is notable that the other APP tested here, M-SAA3 and CRP did not correlate with Hp or SCC or between themselves. Future investigation should examine the cause of the lack of correlation; however as potential biomarkers it may be that the 3 APP are responding to differing stages of mastitis or to differing pathogens and optimal diagnostic value could be obtained by measuring all APP in a multiplexed assay approach. This could even define the stage or pathogen and therapy could be tailored appropriately. Indeed the ultimate use of APP testing could be in automated milking systems where immunoassay based systems for APP quantification could be developed. It is vital therefore that the knowledge base on APP levels in dairy cows is more complete in order to allow full use of their undoubted potential as biomarkers of mastitis.

## Conclusions

In this study a median basal concentration of 3.08 μg/ml, 0.96 μg/ml and 22.4 ng/ml of Hp, M-SAA3 and CRP respectively, were found in CMS from cows free from SM (as defined by low SCC levels of <200,000 cells/ml) and CM (absence of clinical signs) on a small scale commercial dairy. Generally, QMS Hp had higher range than CMS presumably due to dilution effect on CMS.

It would be advantageous to further determine the basal profile of APP in other commercial dairy farms and compare with the findings of this study in order to define reference ranges for mid-lactation or periparturient cows’ milk. This can then be used in influencing the diagnosis of mastitis based on the assay of Hp, M-SAA3 or CRP. In addition, because immunoassays are utilized for measuring APP, the possibility for adopting such tests for use as rapid on-line farm tests exists and can offer advantage in sensitivity, ease of use and timeliness of obtaining results.

## Abbreviations

APP: Acute phase proteins
APR: Acute phase response
CM: Clinical mastitis
CMT: California mastitis test
CMS: Composite milk samples
CRP: C-reactive protein
CV: Coefficient of variation
DIM: Days in milk
EC: Electrical conductivity
IMI: Intramammary infection
IRT: Infra-red thermography
ELISA: Enzyme linked immunosorbent assay
Hp: Haptoglobin
Hb: Haemoglobin
HRP: Horse radish peroxidase
IMI: Intramammary infection/inflammation
LOD: Limit of detection
M-SAA3: Mammary associated serum amyloid A3
NAGase: N-acetyl-β-D-glucosaminidase
NMR: National milk records
QC: Quality control
QMS: Quarter milk samples
Rpm: Revolutions per minute
SAA: Serum amyloid A
SCC: Somatic cell counts
SM: Subclinical mastitis
SPSS: Statistical package for social sciences
TMB: Tetra methyl benzidine
TMR: Total mixed ration
x: Times
